# Factors associated with HIV-status disclosure to HIV-infected children receiving care at Kilimanjaro Christian Medical Centre in Moshi, Tanzania

**DOI:** 10.11604/pamj.2014.18.50.2307

**Published:** 2014-05-15

**Authors:** Livin Peter Mumburi, Bernardus Carolus Hamel, Rune Nathanael Philemon, Gibson Nsokolo Kapanda, Levina January Msuya

**Affiliations:** 1 Kilimanjaro Christian Medical University College, P.O. BOX 2240, Moshi, Tanzania

**Keywords:** Paediatric HIV, HIV disclosure status, parents, caregivers

## Abstract

**Introduction:**

With the introduction of antiretroviral drugs HIV-infected children live longer. Disclosure of HIV diagnosis is increasingly an important and inevitable issue. Both healthcare providers and caregivers face challenges of disclosure to children. The objective of the study was to explore factors associated with HIV-status disclosure to HIV-infected children receiving care at Kilimanjaro Christian Medical Centre (KCMC).

**Methods:**

A cross-sectional hospital-based study was conducted from October 2011 to April 2012. Study population included HIV-infected children aged 5 to 14 years, their caregivers and healthcare providers. Structured questionnaires were used to collect information. Children were asked the reason for hospital visits. Outcome of interest was HIV disclosure status. Data was processed and analysed using SPSS version 16.0. Multivariate logistic regression at 5% margin error was used to account for confounders.

**Results:**

A total of 211 children were enrolled with mean age of 9.7 (SD ± 2.6; range 5-14) years. Only 47 (22.3%) children knew their HIV-status. The mean age of disclosure was 10.6 years. Most of disclosed children were aged above 10 years (p).

**Conclusion:**

Most of children were not disclosed. Ages, self medication, getting other support and parents/caregivers prior discussion were strong predictors of disclosure status.

## Introduction

Worldwide about 2.5 million children younger than 15 years of age are infected with HIV and 90% (2.3 million) of them live in Sub-Saharan Africa. Although the rate of new HIV infections has decreased, the total number of people living with HIV continues to rise because of increased life expectancy of those infected relative to the pre Antiretroviral Therapy (ART) era [1].

By 2009, in Tanzania it was estimated that 1.4 million people (5.7%), including adults and children, were living with HIV and children below 18 years accounted for 10% [1]. Globally, HIV-related deaths among adults have declined by 33% while that of adolescents aged 15 to 19 years have increased by 50% [2].

The increase of HIV services in low-resource settings and introduction of HIV counselling, testing and treatment coupled with ART during pregnancy has resulted in a dramatic drop in the rate of vertical transmission as well as significantly lowered HIV-related morbidity and mortality rate [2, 3]. As a result of the increased maternal survival coupled with introduction of long-term therapy of antiretroviral therapy, many children with HIV experience a less symptomatic early course of the disease and survive to older ages [4].

Consequently, the disclosure of HIV infection diagnosis to a child is becoming inevitable and an increasingly important issue as caregivers and healthcare providers face challenges of disclosure to children. Furthermore children have right to know their HIV-status and thus prevent transmission to others [5, 6].

In disclosing HIV infection to children, the American Academy of Pediatrics recommended that counselling to the caregiver be provided by healthcare providers, be individualized putting into consideration the child's cognitive ability and developmental stage, and that disclosure is an on-going process. It also insisted that adolescents should know their HIV status and be fully informed in order to appreciate the consequences of their health including sexual behaviour and issues around their treatment [7].

The levels of disclosure vary widely in developing and developed countries from as low as 9% to high as 95% but with an average of 29%. Disclosure also seems to vary with age of the children; with older children above 10 years being more likely to be told their HIV status as compared to children who are less than 10 years. Many (82.6%) school-age children have not been disclosed [8–12].

The aim was of the study was to explore factors associated with HIV-status disclosure to HIV-infected children receiving care at Kilimanjaro Christian Medical Centre (KCMC), with the expectation that the findings of this study will facilitate the formulation of relevant counselling strategies to parents/caregivers of HIV-infected children.

## Methods

This was an analytical cross-sectional hospital based-study. The study population comprised of 211 parent/caregiver-child dyads who received care for HIV infection at Child-Centred Family Care Clinic (CCFCC) and 25 healthcare providers working at CCFCC at KCMC, in Moshi, Tanzania. The study was conducted over a seven months period from October 2011 to April 2012. The study participants were HIV-infected children aged 5 to 14 years. Study participants were obtained by convenient sampling. Parent/caregiver aged 20 years and above accountable for that child was included in the study. Structured questionnaires were used to collect information on caregivers, children and healthcare providers-related factors, and their characteristics and perception on disclosure. Different questionnaires were used to collect information from caregivers, children and healthcare providers. Children were asked ‘why do you come to hospital for regular visits? ’, the optional responses were: ‘I don't know, I am told to come, drug refill, for investigations and I am sick’. Other sample question was ‘What is your illness called?’, the optional responses were: ‘I don't know, HIV, a disease other than option 2 above’. All questionnaires were administered by the same principal investigator. To avoid inadvertent disclosure of child's status, children were interviewed in the presence of their caregivers and the caregivers were interviewed in absence of the children. Our outcome of interest was disclosure and non-disclosure of HIV-infection to participating children.

Complete disclosure means that the child knows his/her HIV-status and has been given disease specific information, while partial disclosure means that the child knows that it is sick but not knowing that it is HIV infected. For instance, a child with age related cognitive limitations may be told that it has few and weak soldiers to defend the body because of attacks by ‘bad guys’. Other support means that the child is getting transport costs or school fees from outside the household.

Data was processed and analysed using SPSS version 16.0. Descriptive results were presented in tables and figures. Relationships of variables were tested using the Chi-square test for categorical variables and p-values less than 0.05 were considered significant. Odds ratio was used to describe associations whereas stepwise multivariate logistic regression at 5% margin of error was done to identify the predictors of HIV-status disclosure and to account for confounders.

We obtained ethical approval from Kilimanjaro Christian Medical University College Research and Ethics Review Committee, and consent and assent from parents/caregivers and children.

## Results

Of 211 children involved in the study mean age was 9.7 (SD ± 2.6; range 5-14) years. Of these 100 (47.4%) were female.

### Disclosure status

Based on parents’/caregivers’ report, majority of children (n=112; 53.1%) had their serostatus undisclosed to them while nearly a quarter (n=52; 24.6%) had partial disclosure and only 47 (22.3%) had complete disclosure. Among 47 HIV-infected children who had their serostatus disclosed, the mean age was of 10.6 years (SD ± 1.7; range 6-14), about three quarters of them (74.5%) in the range of 10-14 years.

### Children's demographic characteristics by their disclosure status (Table 1)

Adolescents were more likely to have their serostatus disclosed than pre-adolescent (OR 0.04; 95% CI 0.01-0.10; p<0.001). Pre-school children were less likely to be informed of their HIV-infection than those in school (p=0.001). Children, whose fathers were alive, were less likely have their serostatus disclosed than those whose fathers are not alive (OR 0.4; 95% CI 0.2-0.8; p=0.012). Children, who got support from outside the household, were 2.4 times more likely to have been told their HIV-status than those who did not get support (OR2.4; 95% CI 1.1-5.0; p=0.024).

**Table 1:** Demographic characteristics of children by their disclosure status (n=211)

Variable	Total	Disclosure status		p-value	OR (95% CI)
		Disclosed	Not disclosed		
		n=47	n=164		
		No. (%)	No. (%)		
**Sex**					
Male	111	23 (20.7)	88 (79.3)		
Female	100	24 (24.0)	76 (76.0)	0.568	0.8(0.4-1.6)
**Age (years)**					
Pre-adolescent (5 – 10)	123	4 (3.25)	119 (96.75)		
Adolescent (11 – 14)	88	43 (48.9)	45 (51.1)	<0.001	0.04 (0.01-0.10)
**Educational status**					
Pre-school	29	0 (0.0)	29 (100.0)		
In school	182	47 (25.8)	135 (74.2)	0.001	Undefined
**Father alive**					
Yes	144	25 (17.4)	119 (82.6)		
No	67	22 (32.8)	45 (67.2)	0.012	0.4 (0.2-0.8)
**Mother alive**					
Yes	138	31 (22.5)	107 (77.5)		
No	73	16 (21.9)	57 (78.1)	0.928	1.0 (0.5-2.0)
**Orphanage status of child**					
Orphan	33	8 (24.2)	25 (75.8)		
Not orphan	178	39 (21.9)	139 (78.1)	0.767	1.1 (0.5-2.7)
**Child getting any support**					
**Yes**	39	14 (35.9)	25 (64.1)		
**No**	172	33 (19.2)	139 (80.8)	0.024	2.4 (1.1-5.0)

### Children's clinical characteristics in the disclosed and non-disclosed group (Table 2)

At the time of enrolment into care and treatment, 113 (53.6%) of the children had advanced disease as defined by WHO clinical stage III and IV, but there was no statistically significant difference to those in WHO stage I and II in terms of disclosure status (p=0.955). Though it was at borderline statistically significant (p=0.051), children with CD4 counts of 350 cells/µL or less were twice more likely to have been told their HIV infection compared to those with CD4 counts of more than 350 cells/µL. Children who were taking medication self-supervised were significantly more likely to have been told about their HIV-status compared to those supervised by parents/caregivers or anyone else (OR 33.1; 95% CI 13.9-78.9; p<0.001).

**Table 2:** Child clinical characteristics in disclosed (n= 47) and non-disclosed group (n=164)

Variable	Total	Disclosure status		p-value	OR (95% CI)
		Disclosed	Not disclosed		
		No. (%)	No. (%)		
**WHO clinical stage**					
Stage I + II	98	22 (22.4)	76 (77.6)		
Stage III + IV	113	25 (22.1)	88 (77.9)	0.955	1.0 (0.5-2.0)
**Child's CD4 categories (cells/µ):**					
350 or less	38	13 (34.2)	25 (65.8)		
Above 350	173	34 (19.7)	139 (80.3)	0.051	2.1 (1.0-4.6)
**Duration of clinic attendance (months)**					
Less than 12	17	5 (29.4)	12 (70.6)		
12 and above	194	42 (21.7)	152 (78.4)	0.462	1.5 (0.5-4.5)
**ART regimen**					
On ARV	182	40 (22.0)	142 (78.0)		
Not on ARV	29	7 (24.1)	22 (75.9)	0.795	0.9 (0.4-2.2)
**Duration of treatment (months)**					
Less than 12	27	5 (18.5)	22 (81.5)		
12 and above	184	42 (22.8)	142 (77.2)	0.616	0.8 (0.3-2.2)
**Who supervises medication**					
Child	46	34 (73.9)	12 (26.1)		
Others	165	13 (7.9)	152 (92.1)	<0.001	33.1 (13.9-78.9)

### Disclosure of HIV status by parents/caregivers characteristics (Table 3)

Of 211 parents/caregivers of HIV-infected children, 173 (82.0%) were female. Age ranged from 20 to 81 years with a median (IQR) age of 39 (33-46) years. Majority (n=130; 61.7%) were parents of the infected child. Parents/caregivers whose age was above 35 years, were 1.9 times significantly more likely to disclose the child's HIV-status than those aged between 20 - 35 years (OR 1.9; 95% CI 1.0-3.3; p=0.032). Parents/caregivers who had discussed HIV status disclosure with healthcare providers were more likely to disclose the child's HIV status (OR 4.4; 95% CI 2.2-8.7; p<0.001) than those who did not.

**Table 3:** Disclosure status to children by parents/caregivers characteristics (n=211)

Variable	Total	Disclosure status		p-value	OR (95% CI)
		Disclosed	Not disclosed		
		n=47	n=164		
		No. (%)	No. (%)		
**Sex**					
Male	38	6 (15.8)	32 (84.2)		
Female	173	41 (23.7)	132 (76.3)	0.289	0.6 (0.2-1.5)
**Age (years)**					
20 – 35	80	12 (15.0)	68 (85.0)		
Older than 35	131	35 (26.7)	96 (73.3)	0.048	0.5 (0.2-1.0)
**Education level**					
Up to primary	131	28 (21.4)	103 (78.6)		
Post primary	80	19 (23.8)	61 (76.3)	0.688	0.9 (0.4-1.7)
**Occupation**					
Unemployed	37	6 (16.2)	31 (83.8)		
Employed	174	41 (23.6)	133 (76.4)	0.330	0.6 (0.2-1.6)
**Relationship to the child**					
Parent	130	26 (20.0)	104 (80.0)		
Caregiver	81	21 (25.9)	60 (74.1)	0.314	0.7 (0.4-1.4)
**Monthly income (Tsh)**					
Up to 100,000	134	30 (22.4)	104 (77.6)		
More than 100,000	77	17 (22.1)	60 (77.9)	0.958	1.0 (0.5-2.0)
**Results of HIV test (n=198)**					
HIV infected	132	30 (22.7)	102 (77.3)		
Not HIV infected	66	17 (25.8)	49 (74.2)	0.637	0.8 (0.4-1.7)
**Discussed disclosure with healthcare provider**					
Yes	73	29 (39.7)	44 (60.3)		
No	138	18 (13.0)	120 (87.0)	<0.001	4.4 (2.2-8.7)

### Reasons for partial disclosure and non-disclosure

The 164 parents/caregivers who had partially disclosed or not disclosed the HIV-status to their children gave the following reasons for not doing so: 71 (43.3%) said children were too young to understand, 50 (30.5%) disclosure may cause negative emotional consequences like discrimination, 28 (17.1%) said the child may not be able to keep it secret and 15 (9.1%) did not know what and how to deliver the disclosure message.

### Reasons for HIV-status disclosure

Common reasons for disclosure by the 47 disclosing parents/caregivers included: the child was becoming suspicious due to regular clinic attendances (n=17; 36.2%), child inquiring about illness (n=15; 31.9%), need for the child to understand his/her problem (n=11; 23.4%), child's right to know his/her problem (n=8; 17.0%), child was very sick (n=5; 10.6%), death of parents (n=4; 8.5%) and 2 (4.3%) were compelled to disclose because the child had to start ART.

### Predictors of HIV-status disclosure (Table 4)

Out of seven variables with p values less than 0.05, only four variables had statistical significance after multivariate logistic regression test. Child's age, person supervising medication uptake to the child, support given to the child and discussion of child's disclosure with healthcare providers were the major predictors of HIV- status disclosure to children.

**Table 4:** Predictors of HIV disclosure status (final table after stepwise multivariate logistic regression test)

Variables	p value	95.0% CI for EXP(B)	
		Lower	Upper
Child age	0.015	0.047	0.721
Who supervises medication	0.000	11.366	220.536
Support to child	0.009	1.641	33.713
Discussed disclosure with healthcare provider	0.001	2.563	34.958

### Responsible person to initiate disclosure to the child

Majority of parents/caregivers (n=138; 65.4%) indicated that the parent or caregiver alone should disclose about the illness to the child while 60 (28.4%) preferred healthcare providers to disclose the HIV-status to the child. Only 8 (3.8%) preferred both parents/caregivers and healthcare providers to initiate disclosure to the child and 5 (2.4%) caregivers indicated that anyone can do it.

### Age at which the illness should be disclosed to the child

Forty one parents/caregivers (19.4%) preferred disclosure to happen between 6-9 years, while 170 (80.6%) preferred disclosure to happen between 10-16 years. The median age preferred for disclosure was 11 years (IQR=6-16).

### Healthcare provider's perception on disclosure

Of the 25 interviewed healthcare providers, majority (n=13; 52%) were doctors, who had working experience with HIV-infected children of 3 to 5 years. Others were nurses (n=2; 8%), counsellors (n=3; 12%), pharmacists (n=3; 12%), social worker (n=1; 4%), medical record workers (n=2; 8%) and lab technician (n=1; 4%). Seventeen (68%) believed that the optimal age for general discussion about child's disease is 5 to 9 years; and most of the healthcare providers (n=22; 88%) choose that age for the reason that children develop cognitive maturation. The majority of healthcare providers (n=15; 60%) believed that the age between 10 to 12 years is more appropriate to have specific discussion on HIV and 13 (52%) believed that caregivers should lead disclosure discussion with the help of healthcare providers.

## Discussion

Our finding that 22% of children know their HIV-serostatus is consistent with studies in Ghana (21%) [11] and Ethiopia (17.4%) [12], is higher than the findings reported in South Africa (9%) [10], but lower than the findings reported in Thailand (30.1%) [13], and in California (USA) (43.1%) [14]. The reasons for these variations could be that in South Africa the study population included children of younger age (5 months - 11 years), as opposed to that of our study participants (5 to 14 years), which is considered to be the time of cognitive maturation and hence the right time for disclosure. The different findings in level of disclosure in our study and Thailand is because in our study we confirmed with the caregivers that they really told the child that they have HIV/AIDS as opposed to Thailand where some caregivers told the children that they had the same infection as their mother. The high level of disclosure in United States may be due socio-cultural differences showing higher levels of expressiveness within the family and more intensive child-parent interactions [14], which is not the case in most African countries, including Tanzania.

Mean age of disclosure was 10.6 years which was different from 9.2 years, reported in Thailand [13] and 8.7 years found in Nigeria [15]. The reason for this age in disclosure in our set up may be because of lack of disclosure protocol in our setting and parents/caregivers reluctance to initiate disclosure.

Regarding reasons for not disclosing HIV-status to children, caregivers reported that disclosure did not happen because children were too young or not emotionally mature enough to be conveyed the message on HIV, not being able to keep a secret and it may cause negative emotional consequences like being stigmatized, discriminated, and become psychologically affected. These reasons are consistent with previous findings from Thailand and Nigeria [13, 15].

The large sample size in our study gave us a good picture of what is prevailing in our setting regarding HIV-status disclosure in children. Though we did stepwise multivariate logistic regression to account for confounders we still acknowledge the possibility of close proximity of ‘self-medication and age’ and this result should be interpreted with cautions.

Most of parents/caregivers in our study preferred disclosure to take place when the child is entering adolescence, i.e. at a median age of 11 years and above. These findings are consistent with those reported in South Africa [10], where caregivers perceived that a median age of 11 years is the best time to have general discussion regarding child's illness and 12 years to tell the child specifically about HIV. Similar findings of an average age of 10.8 years were also reported in England [16]. However our findings differ from those in India and Ethiopia [17, 12], which showed that majority of the caregivers wanted disclosure to take place during the mid-teen age (14-18 years). Caregivers who proposed mid-teen age believed that it is the age at which children become emotionally mature enough to cope with chronic disease and need sex education to prevent spread of infection. However, their argument may be risky because by mid teen age they may actually already involve in sexual activities and without knowing their status, risk transmission, and thus the need disclosure to take place before that age.

We found that 65% of parents/caregivers wanted themselves to be responsible for disclosure initiation and process of HIV-status to their children, consistent with findings reported in Thailand and Nigeria [13, 15], where 57% and 63.5% of caregivers respectively believed that it was caregiver's responsibility to disclose the child's HIV-status.

Parents/caregivers who had discussed disclosure with healthcare providers were more likely to disclose HIV-status to their children, similar to findings reported in South Africa [10], where also caregivers who had discussion with healthcare providers on disclosure to their children were more likely to disclose. This may be due to the fact that parents/caregivers who had the opportunity to discuss disclosure with healthcare providers have more skills on handling disclosure to their children.

Children, who knew their HIV status, were more likely to take the pills themselves as compared to those who were not disclosed. Similar findings were reported in Ghana [11]. Disclosed children have been told the reason of taking the medicines and being responsible for taking their medication without caregiver supervision.

Children getting outside household support e.g. for transport costs and school fees, were twice more likely to know their HIV-status than those who do not get such support. Similar findings have been reported in the United States [6]. Most of HIV-infected children were orphans who needed help from other people. In the course of looking for help, the caregiver sees the need to reveal HIV status to the child.

We believe that disclosure is an ongoing process, commensurate with the child's age and maturity. It starts with partial disclosure and becomes in due course complete disclosure. We would like to encourage further studies geared on assessing the psychological aspects, knowledge and long-term sequelae of children in both the disclosed and non-disclosed groups.

## Conclusion

There was a low level of HIV-status disclosure in children. Efforts should be made to develop a disclosure protocol for healthcare providers, and parents/caregivers should be involved in disclosure protocol development. Healthcare providers should be encouraged to have formal discussion and also to provide health education and community sensitization to alleviate stigma and discrimination. Parents/caregivers should be empowered on disclosure process techniques.
